# Nanostructured Dense Collagen‐Polyester Composite Hydrogels as Amphiphilic Platforms for Drug Delivery

**DOI:** 10.1002/advs.202004213

**Published:** 2021-02-18

**Authors:** Xiaolin Wang, Olivier Ronsin, Basile Gravez, Nicolette Farman, Tristan Baumberger, Frédéric Jaisser, Thibaud Coradin, Christophe Hélary

**Affiliations:** ^1^ School of Pharmacy and State Key Laboratory of Quality Research in Chinese Medicine Macau University of Science and Technology Taipa Macao 999078 China; ^2^ Sorbonne Université CNRS, UMR 7574, Laboratoire de Chimie de la Matière Condensée de Paris Paris F‐75005 France; ^3^ Sorbonne Université CNRS Institut des NanoSciences de Paris INSP Paris F‐75005 France; ^4^ Université de Paris Paris F‐75006 France; ^5^ INSERM Centre de Recherche des Cordeliers Sorbonne Université Université de Paris Paris F‐75005 France

**Keywords:** collagen, composites, dense hydrogels, drug delivery system, in situ nanoprecipitation, polyesters

## Abstract

Associating collagen with biodegradable hydrophobic polyesters constitutes a promising method for the design of medicated biomaterials. Current collagen‐polyester composite hydrogels consisting of pre‐formed polymeric particles encapsulated within a low concentrated collagen hydrogel suffer from poor physical properties and low drug loading. Herein, an amphiphilic composite platform associating dense collagen hydrogels and up to 50 wt% polyesters with different hydrophobicity and chain length is developed. An original method of fabrication is disclosed based on in situ nanoprecipitation of polyesters impregnated in a pre‐formed 3D dense collagen network. Composites made of poly(lactic‐*co*‐glycolic acid) (PLGA) and poly(lactic acid) (PLA) but not polycaprolactone (PCL) exhibit improved mechanical properties compared to those of pure collagen dense hydrogels while keeping a high degree of hydration. Release kinetics of spironolactone, a lipophilic steroid used as a drug model, can be tuned over one month. No cytotoxicity of the composites is observed on fibroblasts and keratinocytes. Unlike the incorporation of pre‐formed particles, the new process allows for both improved physical properties of collagen hydrogels and controlled drug delivery. The ease of fabrication, wide range of accessible compositions, and positive preliminary safety evaluations of these collagen‐polyesters will favor their translation into clinics in wide areas such as drug delivery and tissue engineering.

## Graphical abstract

In situ nanoprecipitation of biocompatible and biodegradable polyesters within a preformed‐3D collagen network enables the synthesis of nanostructured dense collagen‐polyester composite hydrogels. Modulation of polyester hydrophobicity and chain length allows for both improved physical properties of collagen hydrogels and controlled drug release. Hence, the as‐obtained composite hydrogels represent a novel platform for drug delivery and tissue engineering applications.

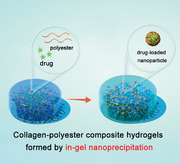

## 1 Introduction

Collagen is the most abundant protein in the mammalian world and benefits from a wide popularity in tissue engineering thanks to its biocompatibility, biodegradability, and capability to promote cell adhesion and proliferation.^[^ ^1^, ^2^ ^]^ It can be applied in the form of film,^[^ ^3^, ^4^ ^]^ hydrogel,^[^ ^5^, ^6^ ^]^ or sponge,^[^ ^7^, ^8^ ^]^ among which hydrogels are of preference in a large range of medical applications due to the moisture‐maintaining capacity and structural similarity to extracellular matrix.^[^ ^1^ ^]^ For instance, collagen hydrogels have been used as tissue engineered devices for the regeneration of skin,^[^ ^9^, ^10^ ^]^ bone,^[^ ^11^ ^]^ cartilage,^[^ ^12^ ^]^ intervertebral disc,^[^ ^13^ ^]^ heart,^[^ ^5^ ^]^ etc. In many reported cases, collagen hydrogels were fabricated from low concentrated solutions and suffered from several limitations such as poor mechanical properties and fast degradability. Another concern lies in encapsulating therapeutic molecules within the hydrogel matrix, which has witnessed growing interest in tissue engineering to favor tissue repair^[^ ^14^ ^]^ or prevent infection.^[^ ^15^ ^]^ Given that many drugs are hydrophobic, low loading and fast drug diffusion were observed due to the poor affinity between therapeutic molecules and collagen network. To circumvent these limitations, dense collagen hydrogels have been developed to lower the hydrogel porosity and increase the network tortuosity, which unfortunately had a limited effect on drug delivery control.^[^ ^16^ ^]^ Therefore, collagen‐based composites with a variety of organic/inorganic materials have been developed. They have been fabricated through different processes, including direct addition of particles/solutions to the collagen solution before gelation,^[^ ^6^ ^]^ deposition of particles into pre‐formed collagen network,^[^ ^17^ ^]^ or by co‐electrospinning a mixture of particles and collagen solutions.^[^ ^18^ ^]^ In a different approaches, few attempts have been carried out by in situ particle formation throughout the 3D hydrogel network.^[^ ^19^ ^]^

Among the materials applied in collagen‐based composites, polyesters have been broadly used. The most investigated ones are poly(lactic acid) (PLA), poly(lactic‐*co*‐glycolic acid) (PLGA), and polycaprolactone (PCL), all of them are approved by the American Food and Drug Administration (FDA) for tissue regeneration due to their satisfying biocompatibility and biodegradability.^[^ ^20^ ^]^ Moreover, they allow for the encapsulation both of lipophilic drugs and hydrophilic drugs including growth factors and nucleic acids by emulsion method.^[^ ^21^, ^22^, ^23^ ^]^ By tuning the polyester nature and particle size, the drug delivery kinetic, which is generally based on diffusion and/or particle erosion, can be tuned.^[^ ^24^ ^]^ Yet, collagen‐polyester composites used in tissue engineering have several drawbacks such as the utilization of surfactants and crosslinkers during the fabrication process. In addition, the polyester particles barely improve the physical properties of low concentrated collagen hydrogels. Hence, a process of fabrication which could enhance the physical properties of collagen based composite materials and allow for the controlled and sustained release of bioactive molecules is of high interest.

**Figure 1:**
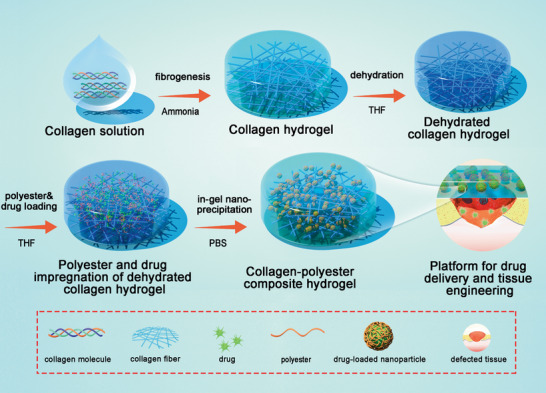
Schematic diagram of fabrication of collagen‐polyester composite hydrogels based on in situ nanoprecipitation process, which has promising applications in drug delivery and tissue engineering.

Nanoprecipitation generally refers to the process which instantaneously generates a dispersion of small droplets or nanoparticles (NP) in the 50–300 nm range without the presence of surfactant.^[^ ^25^ ^]^ In this study we have developed a novel in situ nanoprecipitation method to introduce hydrophobic polyesters in large amounts and in the presence of lipophilic drugs in dense collagen (Col) hydrogels (**Figure** **1**). Compared to most common low‐concentrated hydrogels, dense collagen matrices feature high hydration, improved mechanical properties and extended in vivo stability.^[^ ^16^, ^26^, ^27^ ^]^ Polyesters exhibiting higher rigidity than soft collagen (Table S1, Supporting Information) can reinforce hydrogel matrix upon associating with collagen fibers.^[^ ^28^ ^]^ To the best of our knowledge, this is the first time that polymer nanoprecipitation within a dense hydrogel matrix is reported.

**Figure 2:**
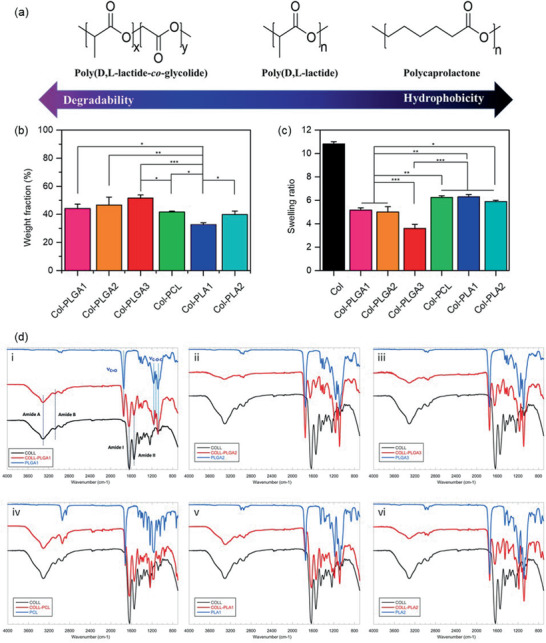
a) Chemical structure of polyesters involved in collagen composite hydrogel preparations. b) Mass fraction of polyesters in lyophilized collagen composites (*n* = 4). c) Swelling ratios of collagen or collagen composite hydrogels after rehydration overnight in PBS 1 × (*n* = 4). Variance among all the groups except for Col group was determined by one‐way ANOVA with Tukey posthoc test (^*^ *p* < 0.05, ^**^ *p* < 0.01, ^***^ *p* < 0.001). d) FTIR spectra of collagen, polymer, and composites: PLGA1 (i), PLGA2 (ii), PLGA3 (iii), PCL (iv), PLA1 (v), PLA2 (vi).

Herein, a platform of collagen‐based composites free of surfactants and crosslinkers was generated using several polyesters encompassing different hydrophobicity and chain lengths (Table S1, Supporting Information). The impact of polyesters (i.e., PLGA, PLA, and PCL; **Figure** **2**a) on composite physical properties, drug delivery kinetic as well as cytocompatibility was assessed in depth. For this purpose, spironolactone, an antagonist against mineralocorticoid receptor (MR) favoring skin tissue repair,^[^ ^29^ ^]^ was chosen as a model drug. The as‐fabricated collagen composite represents a robust paradigm to bolster therapeutic performance by facilitating on‐demand cargo release and providing sufficient mechanical properties for a wide range of biomedical applications.

## 2 Results

### 2.1 Process of Fabrication‐Physical Properties

In this study, composite hydrogels were fabricated by in situ precipitation of polyesters throughout a collagen hydrogel matrix using phosphate buffer saline (PBS) as non‐solvent. In a first step, a concentrated acidic solution of type I collagen (40 mg.mL^−1^) was converted into a fibrillar dense hydrogel by neutralization under ammonia vapor.^[^ ^16^ ^]^ Then, a solvent exchange step was undertaken to replace water by tetrahydrofuran (THF), using THF/H_2_O mixtures of increasing organic content. In a next step, the resulting organogels were immersed in a THF solution containing the polyester and the drug, allowing them to diffuse inside the collagen network porosity. Finally, the aqueous phosphate buffer was added, inducing precipitation of the polymer in a particulate form. At this stage, lipophilic drugs are more likely to precipitate with the hydrophobic polyesters than to remain freely in the water‐filled pores of the collagen network.

According to Figure 2b, the polyester weight fraction in the composites ranged from 32.6 to 51.5 wt%, with Col‐PLA1 at the lowest and Col‐PLGA3 at the highest value. The weight fraction of PLGA and PLA immobilized within dense collagen hydrogel slightly increased with the chain length of the polyester (Figure 2b). Using polymers with a similar molecular weight (*M*_w_) around 15 kDa (PLA1, PLGA1, and PCL), the ability of precipitation within the collagen network was slightly higher for PLGA compared to that for PLA and PCL.

Swelling property of lyophilized collagen composites was evaluated in terms of weight ratio of absorbed water over the polymeric matrix weight. Pure collagen scaffold exhibited the highest swelling properties with a swelling ratio over 10, that is, around 2–3 times that of composite hydrogels. As the collagen mass is the same in all samples, the lower capacity of swelling can be attributed to the higher mass of polyesters which are unable to swell. For example, Col‐PLGA1 was observed with slower water uptake compared with pure collagen, which took 10 and 6 h to reach equilibrium, respectively (Figure S1, Supporting Information). However, differences in swelling properties were observed depending on the polymer used. The innate hydrophobicity of polyesters did not impact on swelling properties. For instance, the different hydrophobic polymers with a low molecule weight had similar effects on the water absorbance. In contrast, the chain length negatively impacted composite hydrogel swelling because the ratio measured for PLGA1 (short chain) decreased from 6 to 3.6 for PLGA 3 (long chain) (Figure 2c).

All composite materials were analyzed by attenuated total reflectance–Fourier transform infrared (ATR–FTIR) spectroscopy after freeze‐drying and compared with pure collagen and polymeralone materials processed in similar conditions (Figure 2d). The spectrum of the composites was, in all cases, a combination of the spectra of the two individual components. As a typical example, Figure 2d‐i shows the detailed comparison of collagen, PLGA1 and Col‐PLGA1 materials. Despite some overlap between protein and polymer vibration bands, the 3300, 3080, 1645, and 1550 cm^−1^ peaks in the spectrum of the composite could be unambiguously attributed to the Amide A, Amide B, Amide I, and Amide II bands of collagen, respectively.^[^ ^30^ ^]^ In parallel the peak at 1750 cm^−1^ and the triplet between 1180 and 1080 cm^−1^ could be assigned to the carbonyl group and ester bonds of the polymer, respectively.^[^ ^31^ ^]^ The relative intensity of Amide I and Amide II bands was similar in pure collagen and Col‐PLGA1 and the peaks of the carbonyl and ester bands were not shifted from PLGA1 to Col‐PLGA1. This indicates that neither the conformation of collagen nor the backbone of the polymer was modified within the composites, suggesting the absence of strong interactions between these two components. Additional analyses (Figure S2, Supporting Information) showed that the spectra of as‐received polymer and THF‐dissolved/buffer precipitated polymer were identical. Finally, it was not possible to use FTIR to unambiguously ascertain the presence of spironolactone, neither in polymer alone nor in composite materials.

### 2.2 Ultrastructure of Collagen‐Polyester Composite Hydrogels

**Figure 3:**
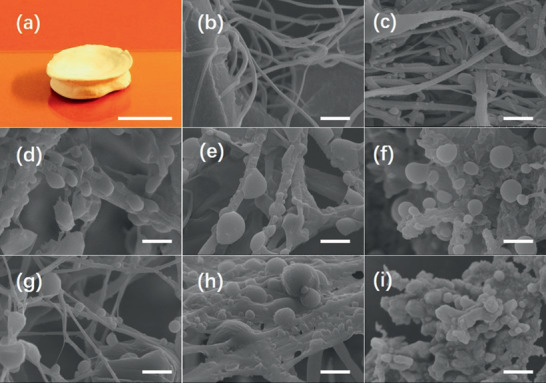
a) Macroscopic view of collagen polyester composites, scale bar 1 cm. b–i) Structure of collagen composites observed by SEM images exhibiting the formation of polyesters nanoparticles along the collagen fibers: Col (b), Col‐PLGA1 (c), Col‐PLGA2 (d), Col‐PLGA3 (e), Col‐PLA1 (f), Col‐PLA2 (g), Col‐PCL (h), and Col‐PLGA1 (i) after collagenase digestion, scale bar = 2 µm.

The typical macroscopic morphology of lyophilized collagen polyester composite was presented in **Figure** **3**a. Microscopically, pure dense collagen hydrogels exhibited a fibrillar network observed by scanning electron microscopy (SEM) (Figure 3b). The in situ precipitation of polyesters led to the formation of particles at the collagen fibrils surface regardless of the polymer studied. The population of PLGA particles was polydisperse in size within composites. The average diameter increased with the PLGA *M*_w_ from 1.20 ± 0.5 µm for PLGA1 to 2.3 ± 0.6 µm for PLGA 3 (Figure 3c–e). The particle size also depended on the polyester nature, with particle size increasing with polymer hydrophobicity. Thus, using polymers with comparable chain length, particles with a diameter of 1.2 ± 0.5, 1.1 ± 0.4, and 2.5 ± 0.5 µm were obtained for PLGA1, PLA1, and PCL, respectively (Figure 3f,h). Diameter of particles observed in composites made with PLA1 and PLA2 (Figure 3f,g) was similar (1.1 ± 0.4 vs 1.2 ± 0.3 µm). Notably, digestion of Col‐PLGA with collagenase (0.1 mg. mL^−1^) resulted in absence of collagen fibrils and only polymeric particles with consistent size observed in the composites (Figure 3i).

Observations by transmission electron microscopy (TEM) revealed the presence of banded collagen fibrils with a 67 nm period in pure collagen hydrogels (Figure S3a, Supporting Information). Similar striated collagen fibrils were also observed in composite hydrogels, indicating the preservation of the collagen ultrastructure regardless of the chemical nature and the chain length of polyesters (Figure S3b–g, Supporting Information). Moreover, polyester particles with submicron size were visible within the collagen network. It is worth noticing that some collagen banded fibrils were located at the polyester surface, circling polymer particles.

### 2.3 Mechanical Properties of Composite Hydrogels

**Figure 4:**
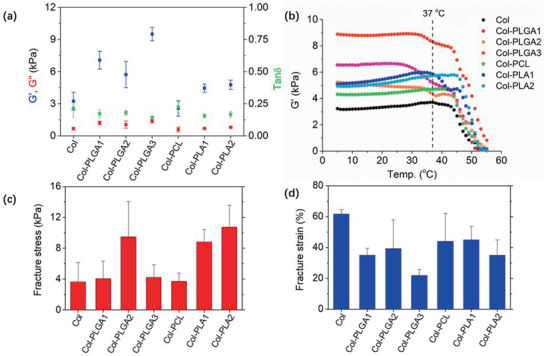
Mechanical properties of collagen composites. a) Shear modulus of collagen composite hydrogels after full rehydration: storage modulus *G*′ and loss modulus *G*″ at 25 °C, *f* = 1 Hz (*n* = 4). b) Temperature dependence of the storage modulus *G*′ of hydrogels tested at *f* = 1 Hz, heating rate at 2 °C min^−1^and strain at 1%. c) Fracture stress and d) fracture strain of hydrogels, obtained by uniaxial tensile test at 25 °C (*n* = 3).

Fibrillar collagen hydrogels are physically cross‐linked, multiscale structured, highly hydrated networks that exhibit characteristic mechanical behaviors of a solid‐like material. Rheological studies were performed under shear oscillatory stress at 25 °C. Each hydrogel studied in this work exhibited a characteristic frequency domain (1–10 Hz) for which the mechanical response was essentially elastic and frequency independent. The value of tan δ = *G*″/*G*′ was always lower than 1 for all hydrogels, indicating the formation of stable 3D hydrogel network (**Figure** **4**a). Storage modulus measured for collagen hydrogels concentrated at 40 mg.mL^−1^ was around 3 kPa as previously shown.^[^ ^16^ ^]^ The stiffness of the composite was always larger than that of pure collagen, except for Col‐PCL for which no significant difference was found. Compared to pure collagen, the storage modulus doubled for Col‐PLGA1 and Col‐PLGA2 and tripled for Col‐PLGA3, which only increased 1.5 times for composites fabricated with PLA.

To meet the demand for biomedical application, composite materials are required to maintain certain level of extensibility while bearing considerable stress prior to fracture. In this regard, the fracture stress and strain of the materials were tested by uniaxial tensile test at ambient temperature (Figure 4c,d). Pure collagen hydrogels could be strained up to 60% of their initial length before breaking and the fracture stress measured was around 4 kPa. Composites formed with PCL exhibited a similar mechanical behavior. The addition of other polyesters within the collagen network reduced the ability of hydrogels to be strained by ≈33%, except for PLGA3. For this composite, the reduction was around 66%. This decrease of deformability was associated with an increase of the fracture stress. The value measured for Col‐PLGA2, Col‐PLGA3, Col‐PLA1, and Col‐PLA2 hydrogels was double that of pure collagen hydrogels. Surprisingly, the fracture stress measured for Col‐PLGA1 was similar to that of pure collagen.

### 2.4 Dynamic Mechanical Thermal Analysis

To have a better understanding of the temperature dependence of collagen composites, rheological measurement of materials was performed from 5 to 55 °C. As shown in Figure 4b, elastic moduli of all hydrogels were barely influenced by temperature change below 28 °C. With the further increase of temperature, collagen and composites started to exhibit distinct profiles. For pure collagen, *G*′ was not impacted by a temperature below 45 °C, revealing that collagen molecules were all self‐assembled into collagen fibrils to form stable 3D matrix. Above this temperature, a sharp decrease of *G*′ of 2 orders of magnitude was observed, indicating the denaturation of collagen.^[^ ^32^ ^]^ Interestingly, the decrease of the storage modulus associated with the collagen denaturation appeared at similar or higher temperature for all composites, suggesting an improved thermal stability of composite hydrogels. Meanwhile, an additional small decrease in *G*′ was noticed for all Col‐PLGA and Col‐PLA1 near 37 °C, evidencing the contribution of the glassy‐to‐rubbery state transition of the polyesters on the mechanical behavior of the composites at physiological temperature.

### 2.5 Drug Loading and Release Kinetics

**Figure 5:**
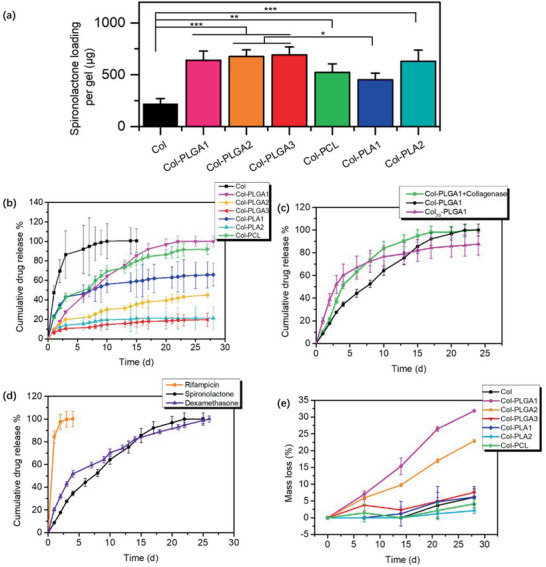
a) Spironolactone loading in pure collagen or the composites (*n* = 3). Variance among all the groups was determined by one‐way ANOVA with Tukey posthoc test (^*^ *p* < 0.05, ^**^ *p* < 0.01, ^***^ *p* < 0.001). b) Drug release kinetics of spironolactone from collagen or collagen composite hydrogels (*n* = 3). c) Drug release kinetics of spironolactone from Col‐PLGA1 with (green, *n* = 3) and without (black) the presence of collagenase, Col_20_‐PLGA1 (pink, *n* = 3). d) Drug release profiles of rifampicin, dexamethasone, and spironolactone from Col‐PLGA1 (*n* = 3). e) Degradation profile of pure collagen and composite hydrogels in PBS at 37 °C (*n* = 3).

Using Spironolactone as a model lipophilic drug, it was possible to evidence that polymer incorporation in the composite hydrogels not only significantly improved drug loading, from *≈*200 µg per gel for pure collagen up to *≈*600 µg per gel for Col‐PLGAs, but also strongly impacted the drug release profiles (**Figure** **5**a,b). Drug loading calculated by weight percentage of spironolactone in the gels was ≈1% for pure collagen and ≈1.6% for collagen composites, respectively while the encapsulation efficiency of spironolactone in pure collagen or the composites was 32.2–49.4% for collagen composites, which was 2–3 times that of pure collagen (Figure S4a,b, Supporting Information).

Pure collagen hydrogels exhibited poor control over spironolactone delivery, as more than 80% of the drug content was released after 3 days with a burst release as high as 50%, which reflects their high hydrophilicity. Regarding the composite hydrogels, remarkably retarded drug release profile could be observed, and the release kinetics was affected by both the chemical structure and *M*_w_ of the polymer. Most striking results were obtained with Col‐PLGA1 and Col‐PCL that showed a sustained release over 1 month. Especially Col‐PLGA1 has the advantage of lower burst release on day 1, which was around 10% of the loading, and corresponded to the fraction of spironolactone that was not encapsulated in the polymer particles but retained in the pores of the collagen network. In parallel, it was noticed that, for the same polymer type, the higher the *M*_w_, the slower were drug release kinetics, in agreement with previous reports.^[^ ^24^ ^]^

In general, diffusion, erosion, and degradation are the major mechanisms involved in drug release from bioerodible drug delivery systems. Based on the above reasoning about collagen structure, molecular drugs (*M*_w_ of 0.5 to 2 kDa, molecular dimensions of 0.5 to 1.5 nm) should not experience hindered diffusion in transport through the hydrogel matrix (estimated mesh size: 58 nm).^[^ ^33^ ^]^ Mathematical modeling of drug release kinetics provides a basis for the study of mass transport mechanisms.^[^ ^34^ ^]^ Zero‐order, first order, and Higuchi models were evaluated and the best ones for each sample are provided in **Table** **1**, together with the corresponding equation and regression coefficient. To allow for comparison between different composites, all curves were fitted with the most popular of the empirical/semi‐empirical mathematical models, that is, the Ritger–Peppas equation,

(1) $$\frac{M_{\infty }}{M_{t}}=\;at^{n}$$

where *M*_t_ is the amount of drug released at time *t*, *M*_∞_ is the total amount of encapsulated drug, *a* is a constant incorporating characteristics of the system and *n* is the release exponent, the value of which may be indicative of the release mechanism. The power *n* depends on the type of transport, hydrogel geometry, and polymer polydispersity: (i) *n* = 0.50, 0.45, or 0.43 for release from slabs, cylinders, and spheres, respectively, in case of pure Fickian diffusion; (ii) *n* = 1.0 when surface erosion dominates the release; (iii) when *n* value falls in‐between, diffusion and erosion have a synergistic effect on the release; (iv) the equation is valid for the first 60% of the fractional release.

**Table 1:** Fitting results of the drug release profiles

Group	Drug release model fitting			*n* value from Ritger–Peppas model fitting
	Type	Equation	*R* ^2^	
Col	First order	*y* = 98.38(1 − exp(−0.64*x*))	0.9951	0.45
Col‐PLGA1	Higuchi	*y* = 26.54*x* ^1/2^ − 16.99	0.9933	0.85
Col‐PLGA2	Higuchi	*y* = 8.35 *x* ^1/2^ + 2.21	0.9862	0.45
Col‐PLGA3	Higuchi	*y* = 3.64 *x* ^1/2^ + 2.63	0.9601	NA
Col‐PCL	Ritger–Peppas	*y* = 22.34*x* ^0.46^	0.9886	0.46
Col‐PLA1	First order	*y* = 56.67(1 − exp(−0.51*x*))	0.9607	0.43
Col‐PLA2	Higuchi	*y* = 5.13 *x* ^1/2^ + 4.06	0.9812	NA
Col‐PLGA1 + collagenase	First order	*y* = 124.59(1 − exp(−0.11*x*))	0.9752	1.05
Col_20_‐PLGA1	First order	*y* = 83.19(1 − exp(−0.29*x*))	0.9945	0.64
Col‐MP	First order	*y* = 175.91(1 − exp(−0.027*x*))	0.9913	0.97
MP	First order	*y* = 114.04(1 − exp(−0.097*x*))	0.9947	0.95
Col‐NP	Higuchi	*y* = 26.91 *x* ^1/2^ − 12.83	0.9897	0.73
NP	First order	*y* = 101.11(1 − exp(−0.17*x*))	0.9878	0.62

NA = not applicable, as the data are not valid for Ritger–Peppas model fitting.

In the present situation, hydrogels have a disc shape and should therefore show a limiting *n* value of 0.45, as confirmed by our fitting procedure (Table 1). For Col‐PLGA 1 composite hydrogels, the release kinetic was constant and sustained for 2 weeks and slightly decreased during the following 2 weeks (Figure 5b). After one month, the entire dose of spironolactone was released. The calculated *n* = 0.85 value showed that drug release was controlled by diffusion from PLGA1 and particle erosion. For PLA1 and PCL, drug delivery was exclusively based on Fickian diffusion from polymers (n ≈ 0.45). Drug diffusion was slower when PLGA or PLA of higher *M*_w_ was used (Figure 5b) and the fitting procedure was less accurate (Table 1). Surprisingly, no clear correlation between drug release kinetic and polymer hydrophobicity was observed as the spironolactone release from PCL was faster than that from PLA. Overall, the composite hydrogels mostly release spironolactone by diffusion and variations in polymer chemistry and *M*_w_ allows for tuning drug loading and release kinetics. With the aim of confirming our model, an in vitro degradation experiment was performed in simulated physiological conditions (PBS at 37 °C) over 28 days. Col‐PLGA1 and Col‐PLGA2 exhibited a linear degradation kinetic reaching *≈*32% and 22% of mass loss, respectively, at day 28 (Figure 5e). The other composites were not prone to erosion, as less than 5% of their initial mass was lost at the end of the experiment. These results were therefore overall consistent with our release model. PLGA2 appears as an intermediate case for which erosion seems to play a role in the release kinetics but this contribution is not predominant and cannot therefore be accurately reflected by usual kinetics models.

To disclose the role played by collagen matrix in the controlled drug release, its simulated enzymatic degradation was undertaken using collagenase (0.1 mg. mL^−1^). Complete digestion of Col was observed after 2 h of incubation at 37 °C. In contrast, when the same procedure was applied to composite hydrogels, large bulks were still visible, giving first evidence that the presence of the hydrophobic polymers considerably improves the stability of the matrix in inflammatory‐like aggressive environments. In parallel, collagenase digestion accelerated spironolactone release from day 3 (Figure 5c, green curve). This should reflect the fact that the partial removal of collagen from the polymer particle surrounding results in a higher exposure to water of its surface as well as to encapsulated drug. This should lead to an accelerated hydrolysis of the polymer and faster diffusion of spironolactone from the matrix to the medium. In fact, the increase in the *n* value from 0.84 to 1.05 suggests that the former effect, that is, easier access of water to particle surface favoring erosion, prevails.

Further insights in the role of the collagen matrix were gained by using a less concentrated protein network (20 mg.mL^−1^ instead of 40 mg.mL^−1^). The resulting Col_20_‐PLGA1 demonstrated a significantly different drug release profile compared to Col‐PLGA1 (Figure 5c, pink curve). From the mechanistic perspective, the decrease in collagen concentration lowered the n value from *n* = 0.85 for Col‐PLGA1 to *n* = 0.64 for Col_20_‐PLGA1, indicating a more significant contribution of drug diffusion to the release profile. In parallel, the spironolactone release was markedly faster, with 80% of the drug released after 2 weeks for Col_20_ composite compared to 3 weeks for Col. It must be pointed out that decreasing collagen concentration resulted in a less compact matrix that allowed for the incorporation of more polymer (58.2 wt% PLGA1 in Col_20_ compared to 44.1 wt% in Col) (Table S2, Supporting Information). Thus, decreasing the collagen concentration should favor water access to polymer particles in a similar way to that after collagenase digestion. It is likely that the presence of collagen fibrils on the particle surface may limit its surface erosion. Altogether, these results demonstrate that collagen plays an active role in the drug release profile by providing protection to PLGA against erosion and constitute a barrier for the drug to diffuse through.^[^ ^35^ ^]^

To explore the range of drugs that could be delivered by these novel platforms, two additional bioactive molecules were studied, dexamethasone and rifampicin, considering that (i) they have distinct hydrophobic characters (Xlog P3 = 1.9 and 4.9, respectively, compared to 2.9 for spironolactone, from PubChem), (ii) they have different biological activities (anti‐inflammatory and antibiotic, respectively), and (iii) both of them have already been encapsulated in PLGA particles. Measured release rates increased in the order Rifampicin >> Dexamethasone > Spironolactone, in good correlation with their solubility in PBS, that is, 1260, 89, and 22 µg.mL^−1^, respectively (Figure 5d). Rifampicin was previously reported to be released at 80% within 1 h from PLGA microspheres (MP) (1–3 µm).^[^ ^36^ ^]^ In our study the release period was extended to 2 days. Accordingly, for dexamethasone, MP prepared with PLGA1 were reported to have a 70% release in 24 h followed by complete release within 10 days.^[^ ^37^ ^]^ Here, the release occurred in two steps, including a relatively fast one in the first 5 days, followed by a secondary zero‐order release step for the next 20 days. Taken together, these data indicate that here‐described collagen composite hydrogels can control the delivery of a wide range of bioactive molecules, but are especially well‐suited for more lipophilic ones.

### 2.6 Comparison with Composites from Pre‐Formed Particles

**Figure 6:**
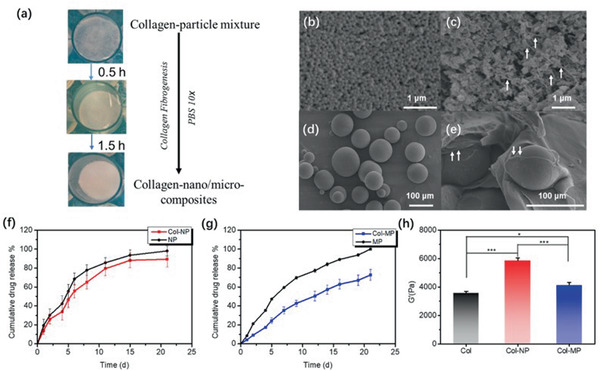
a) Fabrication process of collagen‐nano/microcomposites. SEM images of b) spironolactone‐loaded PLGA1 nanoparticles (NP), c) collagen‐nanocomposites (Col‐NP), d) spironolactone‐loaded PLGA1 microspheres (MP), and e) collagen‐microcomposites (Col‐MP), scale bars were presented in respective figures and white arrows highlighted the interface of collagen and particles at nano/micro scale. Drug release profile of f) NP (black) and Col‐NP (red), g) MP (black) and Col‐MP (red). All groups of drug release were evaluated in triplet. h) Storage modulus *G*′ of collagen‐nano/microcomposites after full rehydration at 25 °C, *f* = 1 Hz (*n* = 4). Variance among all the groups was determined by one‐way ANOVA with Tukey posthoc test (^*^ *p* < 0.05, ^**^ *p* < 0.01, ^***^ *p* < 0.001).

In order to highlight the benefits of the in situ nanoprecipitation strategy disclosed here, spironolactone loaded‐PLGA1 particles, either ≈100 nm (NP) or ≈100 µm (MP) in diameter, were prepared and mixed with concentrated acidic collagen solutions (40 mg.mL^−1^) before neutralization by PBS addition to obtain an alternative type of collagen‐polyester composite (**Figure** **6**a). SEM imaging showed that the particle integrity was not perturbed by the preparation process and that significant collagen‐PLGA interfaces were present within the composite structures (Figure 6b–e).

Overall, these collagen‐PLGA1 composites demonstrated retarded drug delivery compared to particles alone, confirming the previously observed ability of collagen to delay spironolactone release. (Figure 6f,g). More specifically, NP alone exhibited a diffusion‐ and erosion‐controlled drug release behavior for around 10 days (*n* = 0.62) and the corresponding composite (Col‐NP) had a slightly slower delivery rate but comparable mode of release (*n* = 0.73) (Figure 6f). PLGA1 MP alone shows a sustained release over 25 days with a large contribution of erosion‐induced delivery (*n* = 0.95) and the collagen matrix significantly reduced the erosion‐related drug release rate Col‐MP (*n* = 0.97) (Figure 6g).

More accurate comparison between the dissolution profiles of Col‐PLGA1, Col‐MP, MP, Col‐NP, and NP was performed by the similarity factor (*f*_2_),

(2) $$f_{2}=\;50\log\left\{{\left(1+\frac{1}{n}\sum_{t\;=\;1}^{n}{\left(R_{t}-T_{t}\right)}^{2}\right)}^{-0.5}\times \;100\right\}$$

where *n* is the sampling number, *T_t_* and *R_t_* are the percentage of release for the test and reference group at each time point *t*. *f*_2_ factor is 100 when the test and reference profiles are identical, and approaches 0 as the dissimilarity increases.

The *f*_2_ between Col‐NP and NP was 51.4, indicating high similarity in their drug release profile. The *f*_2_ between Col‐MP and MP was 31.2, suggesting that drug release retarding effect of collagen is more significant in microcomposites. The *f*_2_ was higher between Col‐PLGA1 and Col‐NP (52.6) than between Col‐PLGA1 and Col‐MP (39.2) despite the fact that the size of the polymer particles within the in situ formed composite is *≈*1 µm. The closer similarity in release profile was found between Col‐PLGA1 and MP (*f*_2_ = 55.9) whereas Col‐PLGA1 and NP behavior were clearly distinct (*f*_2_ = 37.6). The shear modulus of collagen nano/micro‐composites were also evaluated, showing a significant but small increase in *G*′ upon MP addition whereas the storage modulus doubled in the presence of NP, reaching a comparable value to Col‐PLGA1 (Figure 6h). To summarize, the incorporation of pre‐formed microparticles within collagen hydrogels slow down their spironolactone release but did not improve the matrix mechanical properties. On the contrary, the addition of PLGA NP improved the mechanical properties of the hydrogel but had limited impact on the drug release. Interestingly, Col‐PLGA1 hydrogels seem to bear similarity with both Col‐NP composites and MP particles and thus exhibit both improved mechanical and drug release properties.

### 2.7 Cytotoxicity and Biological Activity of Spironolactone Released from Composite Hydrogels

**Figure 7:**
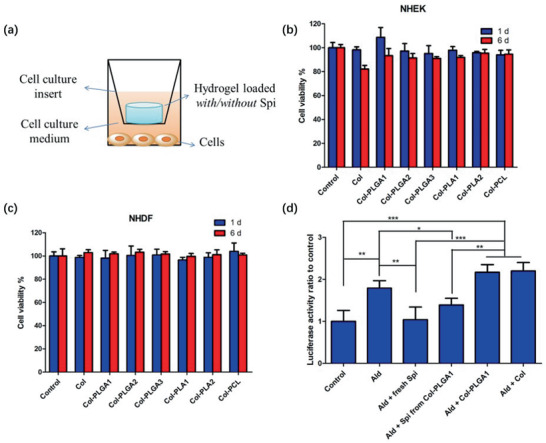
a) Set‐up of cell viability test. Cell viability of b) NHDF and c) NHEK after 1 or 6 day co‐culture with drug loaded collagen or collagen composites (*n* = 3). d) Activity of spironolactone assessed by its capability to inhibit aldosterone/MR‐induced luciferase activity (*n* = 3). Spi and Ald stand for spironolactone and aldosterone, respectively. Variance among all the groups was determined by one‐way ANOVA with Newman‐Keul posthoc test (^*^ *p* < 0.05, ^**^ *p* < 0.01, ^***^ *p* < 0.001).

Type I collagen and PLGA are considered as non‐toxic compounds in regular use. However, it was important to check that the elaboration process did not introduce any harmful substance, such as traces of THF. Assays were performed in a non‐contact mode, allowing to assess the presence of any toxic molecule released from the hydrogel (Figure **7**a). No toxicity toward human skin cells, keratinocytes (normal human epithelial keratinocytes, NHEK) and fibroblasts (normal human dermal fibroblasts, NHDF), was observed for drug‐free collagen and collagen‐polyester hydrogels over one‐week culture (Figure S5, Supporting Information). Another possible risk of toxicity would have come from the high dose of spironolactone released during the initial burst period. However, such a detrimental effect was not detected and cell viability was 100% for all drug loaded materials over the same period (Figure 7b,c). These results were confirmed by the observation of living cells in fluorescence microscopy using a Live/Dead assay. More than ninety percent of cells were alive in each condition regardless of the cell type and the composite studied (Figures S6 and S7, Supporting Information).

Accordingly, it was necessary to verify if the drug activity was impacted by the elaboration process. For this purpose, genetically‐transformed H9C2‐MR‐Luc cardiomyocytes were used, in which expression of the luciferase protein is regulated by the transactivation of the MMTV promoter by the aldosterone/MR complex. Spironolactone being an antagonist of the MR, its presence should inhibit aldosterone‐induced luciferase expression. As shown in Figure 7d, spironolactone released from Col‐PLGA1 composite hydrogel on the last day of effective release exhibited comparable inhibition capability with fresh spironolactone at the same concentration, showing that its molecular structure and drug activity were well preserved during the fabrication process and one‐month drug release. It is worth noticing that release media from unloaded hydrogels were unable to inhibit the production of luciferase, evidencing the specific effect of the drug.

## 3 Discussion

Associating collagen, a natural, hydrophilic bioactive, and biocompatible polymer with synthetic hydrophobic biodegradable polyesters appears as a highly relevant option to design medicated biomaterials such as wound dressings. So far, collagen‐polyester nano/microcomposite hydrogels are generally obtained by mixing powders or suspensions of pre‐formed particles with low concentrated collagen solutions. However, these seldom possess collagen fibrils required for cell attachment and also exhibit limited mechanical properties.^[^ ^35^ ^]^ As collagen solution is acidic, this method also brings in the risk for the hydrolysis of polyesters.^[^ ^38^ ^]^ Moreover, addition of alkaline neutralizing reagents or chemical crosslinkers would result in inhomogeneity of collagen fibrillogenesis and particle distribution as the gelling kinetic would exceed the speed of mixing due to the high viscosity of dense collagen solution. Last, the use of ammonia to form dense collagen nano/microcomposite hydrogels could also jeopardize polyester integrity by hydrolyzing ester bounds. Another popular approach relies on co‐electrospinning into nanofibrous composites,^[^ ^18^, ^39^ ^]^ but preserving collagen innate structure by this technique remains highly challenging. Knitted polyester fibers coated with collagen have been described,^[^ ^40^, ^41^ ^]^ which are resistant but exhibit unsatisfying swelling properties and consequently fail to provide a moist environment. In addition, none of these techniques allows for the preparation of materials with comparable relative amounts of the two components.

The elaboration of materials from collagen and polyesters solutions would, in principle, answer some of these challenges but faces a major issue related to their opposite hydrophilic, respectively hydrophobic, character resulting in the absence of good solvents that are miscible in comparable amounts. In this study, a two‐step process was developed where a dense collagen network is first prepared, whose porosity is used for the following precipitation of polyesters from concentrated solution. With such a process, polyesters are not submitted to degrading conditions of ammonia vapors and collagen fibrillogenesis is not perturbed by the presence of organic solvents and organic polymers. Noticeably, our preliminary attempts relied on the freeze‐drying of the collagen hydrogels followed by impregnation with a THF solution of the polymer. However, because of the poor solvation of the dried collagen fibers by the organic solvent, this approach was not conclusive. We therefore turned our attention to a progressive de‐hydration/re‐solvation approach, as typically performed with ethanol for the preparation of electron microscopy samples.^[^ ^16^ ^]^ This improved both the preservation of the initial collagen network structure but also the reproducibility of the resulting materials.

The nano‐precipitation process, also termed as solvent displacement, involves two miscible solvents, one being a good solvent for the polymer (here THF) and the other one a non‐solvent (here water). When PBS is added to the polyester solution in THF, water molecules can diffuse to reach polymer chains, leading to precipitation of solid particles. Precipitation is favored by the hydrophobicity of polyester, which depends on both its chemical nature and *M*_w_.^[^ ^42^ ^]^ Accordingly, here, the final weight fraction of a given polymer in the composites increases with the *M*_w_ (see PLGA1–3 and PLA1–2 in Figure 2b). However, despite the fact that the hydrophobic character of polyesters evolves as PLGA < PLA < PCL, the weight fraction within the composites follows the PLGA1 > PLA1 ≈ PCL order for comparable *M*_w_ (Figure 2b). This result suggests that the interaction of polyesters with collagen fibers can also impact the precipitation yield. Nevertheless, neither the conformation of collagen nor the backbone of the polymers was modified by the in situ precipitation, as evidenced by FTIR. Therefore, such interactions should be weak, and probably of hydrophobic nature. Along the same line, swelling studies showed that rehydration behaviors of the composites can be correlated with the amount of incorporated polymer but not with the hydrophobicity of polyesters (Figure 2c).

Polyesters were found as nano‐ to micro‐particles well dispersed in the collagen matrix (Figures 3 and 4). This illustrates the fact that, except for the presence of hydrogel matrix, this process resembles the well‐known nanoprecipitation process for polyester nanoparticle preparation.^[^ ^25^ ^]^ However, additional experiments involving direct contact of the polyester solutions with PBS 1 × resulted in fast precipitation of large polymer aggregates instead of micro/NPs (Figure S8, Supporting Information). Polyester concentration and volume ratio between the solvent and non‐solvent have been reported to be the key parameters for the nanoparticle formation.^[^ ^42^ ^]^ As the first parameter is fixed in all composites, it can be suggested that the collagen hydrogel matrix influences the diffusion of water and therefore the local volume ratio between oil and aqueous phases. In addition, TEM imaging allowed for evidencing that some collagen fibrils were present at the surface of the precipitated particles. As a matter of fact, when PBS is added to the THF‐filled material, part of the water is expected to interact with the highly hydrophilic protein network. This not only decreases the quantity of water available to react with dissolved polyester but may also make the fiber surface a preferred site for precipitation.

An important expected contribution of the polymer particles is to improve the mechanical and physical properties of dense collagen hydrogels. Indeed, according to the model of Guth and Gold, it is well plausible to obtain significant enhancement of the matrix stiffness upon addition of particle fillers.^[^ ^43^ ^]^ The amount, geometry, and size of the fillers as well as their molecular interactions with the biopolymer matrix are key parameters determining such an enhancement.^[^ ^44^ ^]^ For instance, highly porous, well‐organized, and homogenously distributed PCL microfibers were found to dramatically increase the elasticity and stiffness of gelatin hydrogel up to 54‐fold compared with pure hydrogel or even PCL microfiber scaffold alone.^[^ ^45^ ^]^ In contrast, PCL increased the *G*′ value of the gelatin matrix at a low level when incorporated in form of MP.^[^ ^46^ ^]^ Here, the incorporation of polyesters resulted in an increase of *G*′ from 3 kPa for pure collagen hydrogels up to 10 kPa for composites. Three key properties of the polymers may be considered: their relative amount within the composites, their intrinsic tensile modulus and their hydrophobicity. The first parameter seems to be of particular relevance as the three higher *G*′ values are obtained for the PLGA series that also show the highest precipitation rate. The two other parameters can be considered comparing PLGA1, PLA2, and PCL that are incorporated in similar amounts. On the one hand, PLGA and PLA have similar reported tensile modulus, while that of PCL is nearly one decade smaller (Table S1, Supporting Information). On the other hand, PLA has intermediate hydrophobicity between PLGA and PCL. Thus, the lower reinforcement effect of PLA compared to PLGA may be attributed to its lower hydrophilicity, while the low intrinsic tensile modulus and high hydrophobic character of PCL lead to a worst‐case scenario in PCL composite. As a matter of fact, electron microscopy images show that PCL particles are bigger and seem to be less distributed along the collagen fibrils compared to other samples. As a result, Col‐PCL mechanical properties are similar to those of pure collagen hydrogels despite of higher polymeric mass.

Temperature‐dependent rheological studies did confirm the highly‐fibrillar state of the collagen dense network. The thermal denaturation event near 45 °C was visible for all composites, supporting our assumption that such a fibrillar organization is not perturbed by the synthetic process of composite formation. In parallel, the Col‐PLGA groups experienced an obvious but small decrease in *G*′ starting from 37 °C, which is reasonably attributed to the glass transition temperature (*T*_g_) of PLGA. More precisely, the higher *M*_w_ of PLGA, the higher transition temperature is required to overpass glass transition within the composite, which is in good agreement with the trend for pure polymers (Table S1, Supporting Information). It is well‐known that dramatic changes in polymer chain mobility take place above *T*_g_ where polymers changes from a hard and brittle “glassy” state into a viscous or rubbery state, resulting in materials softening. However, as the hydrophobic chains are dispersed in the 3D collagen matrix and form discontinuous fillers, the stiffness of materials is only slightly impacted by this transition. The decline of the values below 37 °C would be probably attributed to the hydration of hydrophobic polymers.^[^ ^47^ ^]^ Water would play a role of plasticizer in these conditions. A similar analysis can be performed with the PLA series except that because *T*_g_ of PLA2 alone ranges in the 46–50 °C temperature domain, the transition in Col‐PLA2 probably overlaps with the denaturation process of collagen. Finally, Col‐PCL composites share similar behavior with pure collagen gels as the glass transition temperature (–60 °C) and melting point (59–64 °C) of semi‐crystalline PCL are far from the temperature investigated in our research.^[^ ^48^ ^]^ Altogether, except for PCL, incorporation of polyesters increased the *G*′ modulus of collagen hydrogel and improved their structural stability. Moreover, these composites have an elastic behavior in physiological conditions (37 °C), a property that should be particularly useful in tissue engineering to repair soft tissues such as skin.

Bioactive molecules used in tissue engineering are often antibiotics, growth factors, or anti‐inflammatory molecules. There has been a generally disappointing clinical outcome from growth factors trials due to the rapid diffusion and biodegradation in the wound bed or in the implantation site.^[^ ^49^ ^]^ As a consequence, the treatment requires multiple injections. Macromolecular prodrugs obtained by covalently conjugating drugs to polymer chains is a promising approach to achieve desirable drug delivery or for imaging purpose.^[^ ^50^, ^51^, ^52^ ^]^ Yet it is challenging when dealing with proteins modification. Usually, the implantation site is characterized by inflammation and is filled with proteases.^[^ ^53^ ^]^ Hence, the use of therapeutic molecules not sensitive to proteases such as corticoids is of high interest. Here we selected spironolactone, a drug derived from cholesterol‐like corticoids, that has shown promising therapeutic effect in a variety of disease such as cardiovascular and renal diseases,^[^ ^54^ ^]^ cutaneous chronic wounds,^[^ ^55^ ^]^ age‐related macular degeneration,^[^ ^56^ ^]^ and chorioretinal disorders^[^ ^57^ ^]^ to evaluate the potentialities of the collagen‐polyester composites as platforms for controlled drug delivery.

Polyesters used in this study have been intensively studied as drug release carriers in the forms of NPs, MP, and implants.^[^ ^58^, ^59^ ^]^ Among them, PLGA constitutes the most popular platform due to the possibility to tune its degradation rate by controlling lactic to glycolic acid ratios. PLA is generally used as long‐term drug carriers as release can occur over months even years periods.^[^ ^21^ ^]^ In tissue engineering, PCL is more used as a scaffold material than as particulate drug delivery system due to its slow degradation, that is, as long as 2–3 years.^[^ ^48^ ^]^ Size, geometry, degradation profile as well as interactions between drug and matrix^[^ ^60^ ^]^ are considered as key parameters influencing drug release kinetics, which involved both diffusion and erosion‐based mechanisms.^[^ ^24^ ^]^ Therefore, while incorporation of polyesters within collagen hydrogels aimed at conferring controlled drug delivery properties to the otherwise ineffective protein matrix, it also offered interesting insights on the possible influence of an hydrophilic matrix on the release pathways from hydrophobic particles.

As the test period ranged over 1 month only, it could be expected that drug release would be merely controlled by diffusion, plus a possible contribution of erosion for PLGA, as confirmed by the calculated *n* value from Ritger–Peppas model fitting. The in vitro degradation test in physiological conditions reveals that polymer erosion occurs for Col‐PLGA1 and Col‐PLGA2, starting from day 7 and increasing linearly. As a result, polymer degradation effectively contributes to the drug release for these two composites, but to a minor extent for PLGA2.

Here, as general trend, drug release was the slowest in Col‐PLGA3 and ‐PLA2 and the fastest in Col‐PLGA1 and ‐PCL. This can be interpreted considering that PLGA‐1 and PCL chains are more flexible and should favor water penetration inside the particles, thanks to their *T*_g_ lower than 37 °C.

Furthermore, partial degradation of the collagen matrix of Col‐PLGA1 by collagenase resulted in very sharp shortening of the drug delivery period, which according to the calculated *n* value, was correlated to an increase contribution of the particle erosion to the release. Meanwhile, decreasing collagen concentration in the matrix speed up the diffusion process. This would suggest that the collagen matrix plays a two fold barrier function: it slows down water access to the internal structure of the particles, and thus drug diffusion, and it protects the surface of these particles, preventing their erosion.

Additional experiments performed with pre‐formed nano‐ and micro‐particles of PLGA1 also evidenced that the presence of the collagen matrix was more effective in slowing down drug release for the largest particles. However, only NPs could improve the matrix stiffness. SEM images suggest that the first effect is due to the fact that collagen fibers can fully embed the microparticles while NPs rather appear deposited on protein fibers. In such configurations, only nanosized objects can constitute local cross‐linking points for the collagen network. As the Col‐PLGA1 composite is characterized by a particle polydispersity ranging from nanometric to micrometric sizes, this could explain the concomitant enhancement of the mechanical properties and prolonged drug release.

A last possible role of the collagen network is to retard the release of the drug in the medium as it has to diffuse through the porosity of the hydrogel. As a matter of fact, we have previously shown that increasing the concentration of pure collagen hydrogel resulted in slower drug release.^[^ ^16^ ^]^Here, using two other drugs with higher and lower lipophilicity than spironolactone, we obtained a notably retarded release profile for both of them compared with microparticulate cargos prepared with the same PLGA type in the literature.^[^ ^36^, ^37^ ^]^ Therefore the collagen matrix may also contribute to delay the overall release rate by slowing down the drug transport from the particles to the targeted environment. This is in agreement with a previous report showing that incorporation of dexamethasone‐loaded PLGA NPs within alginate hydrogel matrix resulted in remarkably slowed release.^[^ ^61^ ^]^

Going from a functional material to a biomaterial prototype first involves a series of important in vitro tests. First, the question of safety must be addressed. The maximal concentration of spironolactone used in this study was 10^−2^ m which is not toxic on human keratinocytes and fibroblasts according to preliminary data. However, a change in polymer nature, content or preparation method can be a source of toxic products or by‐products. Cell mortality is the first step to assess toxicity toward main cells present at the implantation sites. Favorably, both collagen and the composites exhibited satisfactory cytocompatibility. A further evidence for the as‐established composites as validate drug delivery system was that the activity of spironolactone was well preserved over one month as attested by luciferase inhibition test. Overall neither the starting materials nor the chemical and physical steps involved in the here‐disclosed process have any negative impact on cell viability and spironolactone therapeutic activity.

## 4 Conclusions

A new family of composites applicable as drug delivery platforms for tissue engineering has been prepared by combining dense collagen hydrogels and FDA‐approved hydrophobic polyesters in an in situ nanoprecipitation strategy. Compared to previous reports, the here‐disclosed process occurs in non‐denaturing/non‐degrading conditions and allows achieving high polymer and drug loadings. Moreover, the resulting materials shows no cytotoxicity against human skin cells, and the biological activity of spironolactone, a steroid with a broad range of therapeutic applications, is preserved during the encapsulation procedure. The incorporated polyesters not only confer offer a tunable delivery rate up to 1 month to collagen hydrogels but also improve their mechanical stability.

In these systems, the collagen matrix plays multiple roles, not only influencing the polymer particle size but also controlling the drug release profile, thanks to its hydrophilic character. Because this methodology involves a pre‐formed hydrogel matrix, it could be amendable to many other biopolymers with different physical, chemical, and biological properties such as chitosan or alginate (Figure S9, Supporting Information). In parallel, these platforms are able to deliver other lipophilic drugs favoring tissue repair, for each of which an optimal polyester host may be identified. Last, but not least, as no chemical modification of the starting materials is required, large‐scale and low‐cost manufacturing can be reasonably envisioned.

## 5 Experimental Section

##### Preparation of Dense Collagen‐Polyesters Composite Hydrogels by In Situ Nanoprecipitation

Collagen solution concentrated at 40 mg.mL^−1^ was obtained by slow evaporation of diluted collagen solution 4 mg.mL^−1^ in 0.5 m acetic acid under sterile conditions (flow bench). Pure collagen hydrogel was prepared under ammonia vapor. Specifically, 0.5 g collagen solution (40 mg.mL^−1^) per well was deposited into a 24‐well plate and centrifuged at a speed of 3000 rpm for 15 min to remove bubbles and obtain a flat surface. The as‐obtained collagen solution was then subjected to ammonia vapor overnight to allow collagen fibrillogenesis which resulted in a white disc of hydrogel. Subsequently, dense collagen hydrogels were washed in several fresh PBS baths for 2–3 days until the pH reached 7.4.

Composite hydrogels consisting with collagen and different types of polyesters, namely PLGA 1–3 (RG 502 H, 75:25, *M*_w_ 7000–17 000; RG 503 H, 75:25, *M*_w_ 24 000–38 000; RG 504 H, 75:25, *M*_w_ 38 000–54 000, Sigma Aldrich), PLA 1–2 (Poly(D,L‐lactide), *M*_w_ 10 000–18 000 and 18 000–28 000, respectively, Sigma Aldrich), and PCL (average *M*_w_ ≈ 14 000, Sigma Aldrich), were prepared by in situ nanoprecipitation. For this purpose, above‐described dense collagen hydrogels were progressively dehydrated in water/THF baths of increasing organic solvent content (50, 70, 90, 95, and 100 vol% of THF) for 1 h each. Then the dehydrated hydrogels were immersed in a polymer solution (160 mg.mL^−1^) in THF, containing spironolactone at 4.2 mg.mL^−1^ for drug‐loaded ones. After 24‐h incubation at room temperature, hydrogels were first rinsed 3 times with pure THF for 15 s followed by 3 rinses in PBS for 0.5 h and finally freeze‐dried overnight.

##### Fabrication of Collagen‐PLGA 1 Composites from Pre‐Formed Particles

PLGA with a LA:GA 50:50 and *M*_w_ 7–17 kDa (RG 502 H, PLGA 1), polyvinyl alcohol (PVA, Mowiol 4‐88) and organic solvents were acquired from Sigma Aldrich (Schnelldorf, Germany). Spironolactone‐loaded PLGA NPs and MP were fabricated using an oil‐in‐water emulsion solvent evaporation technique.^[^ ^62^, ^63^ ^]^ For MP, oil phase was first prepared with spironolactone (8 mg) in 1 mL of PLGA1 dissolved in methylene chloride (10% w/v), subsequently emulsified in a 100 mL aqueous solution of PVA (1% w/v) at 500 rpm and maintained under constant stirring for 3 h to allow MP hardening and solvent evaporation. Recovered particles were then washed with distilled water and collected by filtration. For NPs, 50 mg of PLGA1 and 2 mg spironolactone were dissolved in 5 mL acetone. The polymer‐drug solution was then injected into 100 mL of 1% PVA solution at a flow rate of 20 mL.h^−1^ using an autoinjector under magnetic stirring at 500 rpm. Resulting NPs were collected by centrifugation at 20 000 *× g* for 30 min and washed three times with distilled water. Both NP and MP were lyophilized and kept at −20 °C in dry conditions.

20 mg of as‐obtained particles (NP or MP) and 0.5 mL collagen solution (40 mg. mL^−1^) were precisely weighed and deposited in the same well of 24 well‐plate, mixed mechanically, and centrifuged at 3000 rpm for 15 min. 1 mL PBS 10 × was applied on‐top of the mixture to induce the fibrillogenesis of collagen and avoid NP/MP hydrolysis. After 1.5 h, the as‐formed collagen nano/micro‐composites, denoted as Col‐NP and Col‐MP, were collected, freeze‐dried, and kept at −20 °C before use.

##### Polyester Mass Fraction and Swelling Ratio of Collagen Composite Hydrogels

The polyester mass fraction and swelling ratio of hydrogels were both measured at room temperature using a gravimetric method.

The weights of lyophilized composite gels and pure collagen gels prepared from the same batch of collagen solution were precisely measured on a balance and the value is denoted as *W*_d_ and *W*_c_, respectively. The mass fraction of polyesters incorporated in the composite hydrogel were calculated by the equation as follows:

(3) $$\mathrm{Polyester}\;\mathrm{mass}\;\mathrm{fraction}\left(\%\right)=\left(W_{d}-W_{c}\right)/W_{d}\times 100\%$$

The lyophilized samples were fully swollen in PBS 1 ×, taken out and weighed on a balance after removing the surface water with filter paper, this weight of which was denoted as *W*_s._ Finally, the swelling ratios were calculated by the following equation:

(4) $$\mathrm{Swelling}\;\mathrm{Ratio}=\left(W_{s}-W_{d}\right)/W_{d}$$

##### Fourier Transform Infrared Spectroscopy (FTIR)

ATR–FTIR spectra were recorded on freeze‐dried composite hydrogels using a Perkin Elmer Spectrum 100 equipment. Samples were deposited on the diamond crystal and pressed to optimize signal intensity. For each spectrum, 64 scans were collected between 400 and 4000 cm^−1^.

##### Scanning Electron Microscopy (SEM) and Transmission Electron Microscopy (TEM)

Collagen or composite hydrogels were fixed using 3.63% glutaraldehyde in a cacodylate/saccharose buffer (0.05 m/0.3 m, pH 7.4) for 1 h at 4 °C. For SEM observation, samples were washed three times in a cacodylate/saccharose buffer (0.05 m/0.3 m, pH 7.4) following fixation and dehydrated through successive water/ethanol baths of increasing alcohol concentration from 70 to 100 vol%. Thereafter, samples were dried in a critical point dryer and gold sputtered (20 nm) for analysis. Samples were observed with Hitachi S‐3400N SEM operating at 10 kV. For TEM, samples were post‐fixed with osmium tetroxide in cacodylate/saccharose buffer, washed with fresh cacodylate/saccharose buffer, dehydrated in successive ethanol baths from 50 to 100 vol%, then in ethylene oxide, and finally embedded in araldite. Embedded samples were sectioned on an Ultracut Reichert Jungas before observation. Sections were then observed with a Cryo‐microscope Tecnai spirit G2 electron microscope operating at 120 kV. For each hydrogel, photos were taken by a CCD camera (Orius Gatan 832 digital) and analyzed.

##### Mechanical Properties—Rheological Measurements

Shear oscillatory measurements on wet collagen and composite hydrogels were performed on a rheometer (Anton Paar) equipped with a plane acrylic 24.9 mm diameter geometry. Both base and geometry surfaces were rough in order to avoid sample slipping during measurement and all tests were performed at 25 °C. Mechanical behaviors, namely storage moduli *G*′ and loss moduli *G*″ versus frequency (1–100 Hz), were recorded at an imposed 1% strain, which corresponded to non‐destructive conditions. Before each test, the gap between base and geometry was chosen when a slight positive normal force was applied on gels during measurement. Samples of all groups of collagen hydrogels were tested after overnight swelling.

The dynamic rheology analysis as a function of temperature was performed with 25 mm diameter samples on Anton Paar apparatus. Before testing, the thickness of each sample was measured using a micrometer. The measurements were carried out with a compression mode at a frequency of 1 Hz, a heating rate of 2 °C.min^−1^ and a temperature range from 5 to 55 °C with a chamber deposited over the samples for good temperature control. The set strain was 1% and the applied normal force was 0.4 N. For these experiments, all hydrogels were prepared with 2.5 cm in width and 1.5 mm in thickness. Briefly, 1 g collagen solution (40 mg.mL^−1^ in acetic acid) was transferred in a syringe and centrifuged at 3000 rpm for 5 min to remove the bubbles. Subsequently, collagen was slowly extruded onto the glass to shape the collagen as a ball without any bubble. After depositing the spacers with a thickness of 1.5 mm around the collagen, another piece of glass was covered over the surface and pressed with adequate strength to get a collagen disc, which was then exposed to ammonia vapor overnight. The as‐obtained concentrated collagen gel was washed with PBS 1 × several times until the pH reached 7.4. Then the polymers were introduced following the same procedure as described in Section 1.

##### Mechanical Properties—Uniaxial test

Uniaxial tensile experiments were performed at 25 °C in air with humidifier using a BOSE ElectroForce 3200 Series II Test Instrument. Collagen and the composite gels were used at swelling equilibrium state by immersing corresponding gels in PBS overnight. Afterward, strips with 2 mm in thickness and 2 mm in width were cut from the gels, the two ends of the which were glued to the stainless‐steel crossheads with a cyanoacrylate adhesive drop. Samples and crossheads were immersed in a glass petri dish filled with PBS buffer. Both ends of the test piece were pulled apart at a constant velocity of 100 mm.min^−1^. The fracture stress and the fracture strain were defined as the nominal stress and strain at breaking point, respectively and Young's modulus was defined as the slope of the initial stress–strain curves.

##### Drug Loading of Collagen Composite Hydrogels

To analyze the drug loading, the composite hydrogels were first cut into small pieces and immersed in 1 mL collagenase solution (2 mg.mL^−1^) at 37 °C, centrifuged, and the supernatant and sediments were subject to different treatment before UV detection. For the supernatant, collagenase was precipitated by acetonitrile at a volume ratio of 4:1 to obtain transparent solution (drug amount in this part is denoted as *Q*_s_); for the sediments, they were lyophilized and solubilized with acetonitrile (drug amount in this part is denoted as *Q*_r_). Subsequently, 1 mL of the as‐obtained suspension was taken and mixed with 9 mL of milli‐Q water to precipitate PLGA, 1 mL of which was then mixed with another 9 mL of mili‐Q water for dilution and filtered. Drug loading was calculated by the equation as follows:

(5) $$\mathrm{Drug}\;\mathrm{loading}\left(\mathrm{expressed}\;\mathrm{as}\;\mathrm{mg}\;\mathrm{per}\;\mathrm{gel}\right)=Q_{s}+Q_{r}$$

The amount of spironolactone was measured with UV spectrometer at a wavelength of 244 nm and calculated using a linear standard curve (0.5 to 50 µg.mL^−1^).

##### Drug Release from Collagen Composite Hydrogels

In vitro drug release kinetics of collagen and composite hydrogels were investigated in PBS (10 mm, pH 7.4) over 1 month. Composite hydrogels were submerged in 5 mL of PBS (pH 7.4) in a 50 mL centrifuge tube and then incubated in water bath at 37  °C. Care was taken that sink conditions were achieved for each evaluated drug. At each time point, the release medium was collected and replaced by 5 mL of fresh PBS (pH 7.4). The released drug at each point was measured by UV spectrophotometry.

Accelerated drug release with collagenase was studied to better understand the role of collagen in the drug release profile. To this aim, Col‐PLGA1 composite hydrogel was placed in contact with a collagenase solution (0.1 mg.mL^−1^). Col and Col‐PLGA1 together with 1 mL of collagenase solution were respectively deposited in a dialysis bag (cut‐off of 3 kDa). The release study was carried out over 1 month and the released spironolactone was quantified using above‐mentioned methods.

Weight fraction of drug released with time follows a power law relationship. For all groups, % cumulative drug release (% *M*) was fitted to the following kinetic equations: (i) Ritger–Peppas: plotted as log of % *M* versus log time, (ii) zero order: plotted as % *M* versus time, (iii) first order: plotted as log % *M* retained versus time, and (iv) Higuchi, plotted as % *M* versus square root of time, the corresponding equations were listed as follows:

**Table:** 

Ritger–Peppas	$\frac{M_{\infty }}{M_{t}}=\;at^{n}$	(i)
First order	$M_{\infty }=M_{t}\;e^{-k_{1}t}$	(ii)
Zero order	*M_t_* = *K*_0_ *t*	(iii)
Higuchi	$M_{t}=k_{H}\;\sqrt{t}$	(iv)

where *M_t_* is the cumulative amount of drug released at time *t*, *M*_∞_ is the total amount of drug in the matrix, *K*_0_ is the zero‐order rate constant, *K*_1_is the first order release constant, and *K*_H_ is the Higuchi model‐based release constant. The regression coefficient (*R* ^2^) values obtained in various models were compared to get the best fitted kinetic model.

##### In vitro Biodegradation of Collagen Composite Hydrogels

Pure collagen or composite hydrogels were frozen in liquid nitrogen, lyophilized, and measured for initial dry weight (*W*_i_). Afterward, the as‐obtained dry gels were immersed in 5 mL of PBS (pH 7.4) at 37 °C for reswelling and hydrolytic degradation, with medium refreshed every 7 days over one month. At each time point, the respective hydrogels were frozen again in liquid nitrogen and freeze dried to obtain the degraded dry weight (*W*_t_). The percentage of mass loss at each time point was calculated by (*W*_i_ − *W*_t_)/*W*i × 100%.

##### Toxicity of Collagen Composite Hydrogels

The toxic effect of collagen‐polyester composites was analyzed over a 1‐week period. NHDF (Sigma Aldrich) were cultured in complete culture medium (DMEM, Glutamax, 10% Fetal Bovine Serum, 1% Streptomycin/Penicillin) at 37 °C in a moist atmosphere with 5% CO_2._ Primary NHEK (Promocell) were cultured in keratinocytes growth medium 2 (Promocell) supplemented with CaCl_2_. Tissue culture flasks (75 cm^2^) were kept at 37 °C in a 95% air:5% CO_2_ atmosphere. Before confluence, cells were removed from culture flasks by treatment with 0.1% trypsin and 0.02% EDTA. Cells were rinsed and suspended in the appropriate culture medium before use. For all experiments, cells were plated in six‐well plates at 2 × 10^5^ cells mL^−1^. All experiments were repeated at least three times

Collagen composite hydrogels with a diameter of 0.8 cm were prepared in 48 well plates, following the same procedure described in the previous section. The as‐obtained hydrogels were cast within cell culture inserts to avoid direct contact with fibroblasts or keratinocytes seeded beneath (Figure 7a). Cell viability was first evaluated by Alamar Blue assay at day 1 and 6. For this purpose, inserts containing Col‐PLGA1 composite hydrogels were removed and cells were incubated with 0.3 mL of Alamar Blue solution (10 v/v% in complete medium) at 37 °C for 4 h. Afterward, the medium was collected and diluted with Mill‐Q water to a final volume of 1 mL and the absorbance was measured at *λ* = 570 and 600 nm. Cell viability was calculated and reported as a percentage of the control group, that is, cells without incubation with composites (*n* = 3).

Using the same experimental set‐up, the fraction of dead cells was determined in each condition by fluorescence microscopy. For this purpose, a Live/Dead assay (Invitrogen) was performed. After 24 h incubation with composites, human epidermal keratinocytes or dermal fibroblasts were washed with 1 mL of PBS and treated with a solution of Calcein AM and Ethidium homodimer‐1 according to the manufacturer's instructions. The cells were incubated at 37 °C for 20 min, then washed 3 times with PBS for 5 min and observed with a Zeiss Axio D1 Imager fluorecence microscope. For each condition, pictures from five microscopic fields were taken. The percentage of dead cells was then calculated on each image and the results expressed as the mean ± SD (*n* = 5).

##### Activity of spironolactone released from composites

The biological activity of spironolactone was measured by its ability to inhibit the activation of a Luciferase reporter gene by the aldosterone/MR complex in transformed H9C2‐MR cardiomyocytes.^[^ ^64^ ^]^ The expression of the Luciferase reporter gene was driven by the MMTV regulatory sequences that is transactivated by the aldosterone‐MR complex. Transformed H9C2‐MR‐Luc cardiomyocytes were seeded into 6 well plates at a density of 2 × 10^5^ cells.mL^−1^ per well and cultivated overnight. The following day, the supernatants containing spironolactone collected from Col‐PLGA1 composite hydrogels on the last day of effective release was diluted to a spironolactone concentration of 10^−6^ m before adding to cells. Then, aldosterone at 10^−8^ m was added into wells. Cells were incubated at 37 °C for 24 h and culture media were then collected. The luminescence was measured in each well and normalized by the quantity of proteins, as assessed by a Bradford Assay. The arbitrary value 1 was given to the control samples (without aldosterone addition). The ability to activate the production of Luciferase by aldosterone was calculated by the ratio of normalized luminescence measured in wells treated with aldosterone over normalized luminescence in control samples. Last, the ability of spironolactone to inhibit the formation of the aldosterone/MR complex was evaluated. For this purpose, the ratio of normalized luminescence measured in wells treated with aldosterone + spironolactone over normalized luminescence in control samples was estimated. Cells treated with fresh spironolactone and aldosterone were used as positive control.

##### Data Analysis

Statistical analysis and graphs were processed with Graphpad Prism and Origin. All data were expressed as mean ± standard deviation and analyzed with one‐way ANOVA test followed by Tukey's post hoc analysis unless otherwise indicated. A value of *P*  <  0.05 was considered statistically significant.

## Conflict of Interest

The authors declare no conflict of interest.

## Supporting information

Supporting Information
