# Supplementary material for: Nanostructured Dense Collagen‐Polyester Composite Hydrogels as Amphiphilic Platforms for Drug Delivery
Source: Adv Sci (Weinh). 2021 Feb 18;8(7):2004213. doi: 10.1002/advs.202004213 (PMC8025010; doi:10.1002/advs.202004213)
Supplement: Supplementary file 1 — Supporting Information [file ADVS-8-2004213-s001.pdf]

© 2020 Wiley-VCH GmbH

ADVANCED SCIENCE
Open Access

Supporting Information

for *Adv. Sci.,* DOI: 10.1002/advs.202004213

Nanostructured dense collagen-polyester composite hydrogels as amphiphilic platforms for drug delivery

*Xiaolin Wang, Olivier Ronsin, Basile Gravez, Nicolette Farman, Tristan Baumberger, Frédéric Jaisser, Thibaud Coradin, and Christophe Hélary**

Electronic Supplementary Information

Nanostructured dense collagen-polyester composite hydrogels as amphiphilic platforms for drug delivery

Wang Xiaolin[a,b]*, Olivier Ronsin*[c,d]*, Basile Gravez*[e]*, Nicolette Farman*[e]*, Tristan Baumberger*[c,d]*, Frederic Jaisser*[e]*, Thibaud Coradin*[b] *and Christophe Hélary*[b]

[a] *School of Pharmacy and State Key Laboratory of Quality Research in Chinese Medicine, Macau University of Science and Technology, Taipa, Macau, China.*

[b] *Sorbonne Université, CNRS, UMR 7574, Laboratoire de Chimie de la Matière Condensée de Paris, F-75005 Paris, France.*

[c]*Sorbonne Université, CNRS, Institut des NanoSciences de Paris, INSP, F-75005 Paris, France.*

[d]*Université de Paris, F-75006 Paris, France.*

[e]*INSERM, Centre de Recherche des Cordeliers, Sorbonne Université, Université de Paris, Paris, France.*

Table S1. Physical properties of polyesters involved in collagen composite hydrogel

Polyesters	Molecular weight (kDa)	Tensile modulus (GPa)	Tensile strength (MPa)	Ultimate strain (%)	T_m (°C)	T_g (°C)
PLGA1	7-17					39.5#
PLGA2	24-38	1-4	41-55	2-10	——	40.2#
PLGA3	30-60					41.8#
PCL	1.4	0.2-0.4	20-42	300-1000	59-64	-60
PLA1	10-18	1-3.5	27-50	2-10	——	38-42*
PLA2	18-28					46-50*

Data in columns 3~7 extracted from literature[1] and tensile modulus, tensile strength and ultimate strain were obtained at room temperature, #test in the lab, *data from the manufacturer.

T_m = Melting temperature, T_g = Glass transition temperature.

Table S2. PLGA1 loading in C_{20}-PLGA1 composite

Weight of lyophilized C_{20} (mg)	Weight of lyophilized C_{20}-PLGA 1 (mg)	Mass fraction of PLGA1 (wt%)
14.6±0.4	35.1±1.4	58.2±2.7

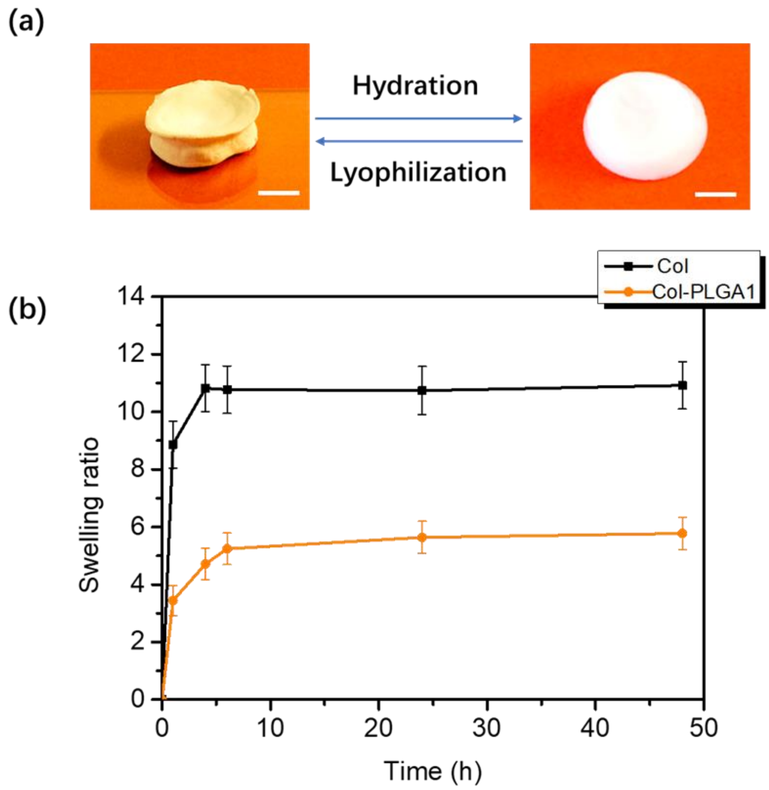

Figure S1. (a) Morphology change of Col-PLGA1 before and after swelling, scale bar = 0.5 cm. (b) Swelling kinetics of Col and Col-PLGA1 over 48 h.

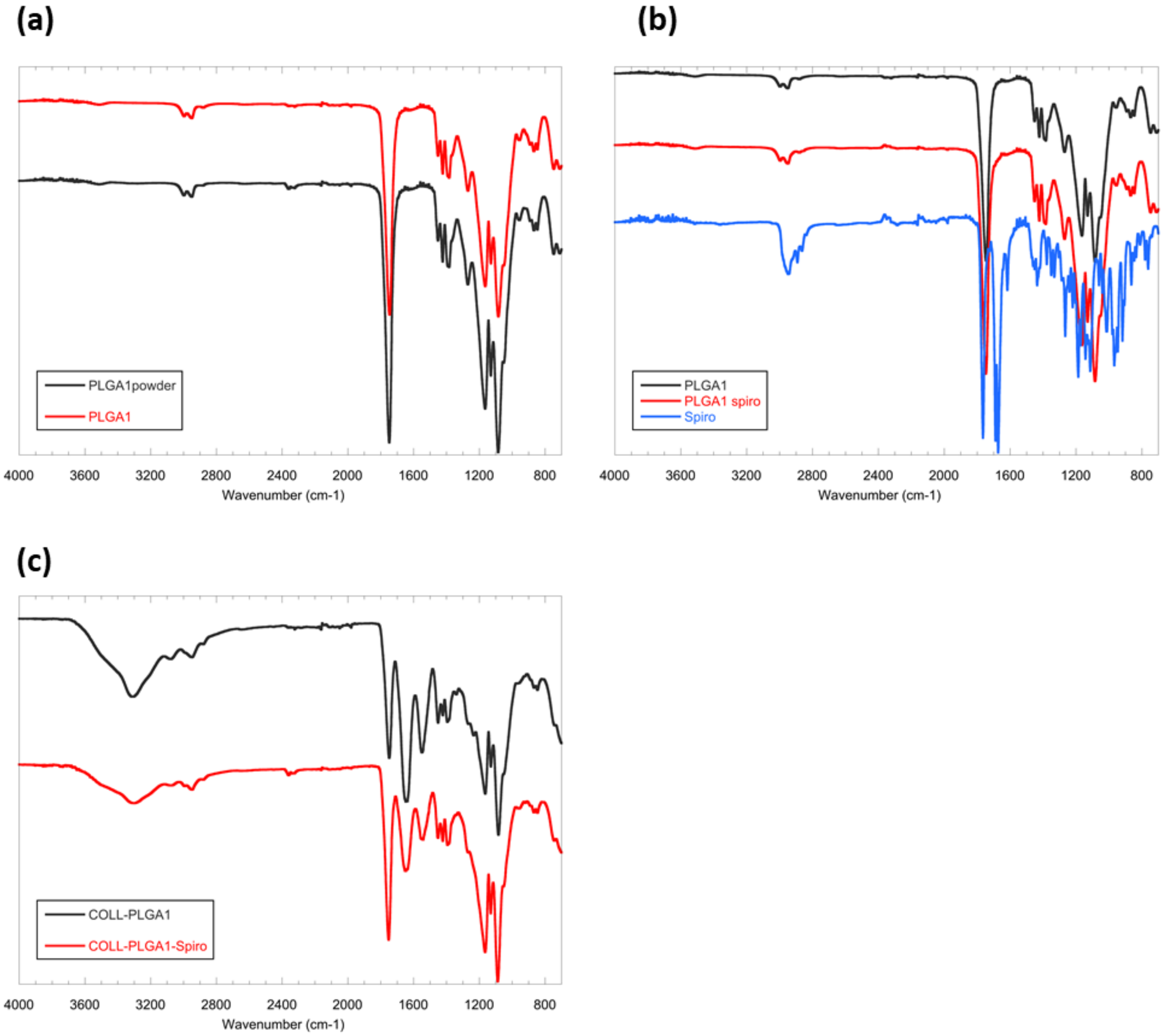

Figure S2. ATR-FTIR spectra of (a) as-received PLGA1 and PLGA1 dissolved in THF and precipitated with buffer ; (b) spironolactone, spironolactone-loaded PLGA1 and PLGA1; (c) unloaded and spironolactone-loaded Col-PLGA1.

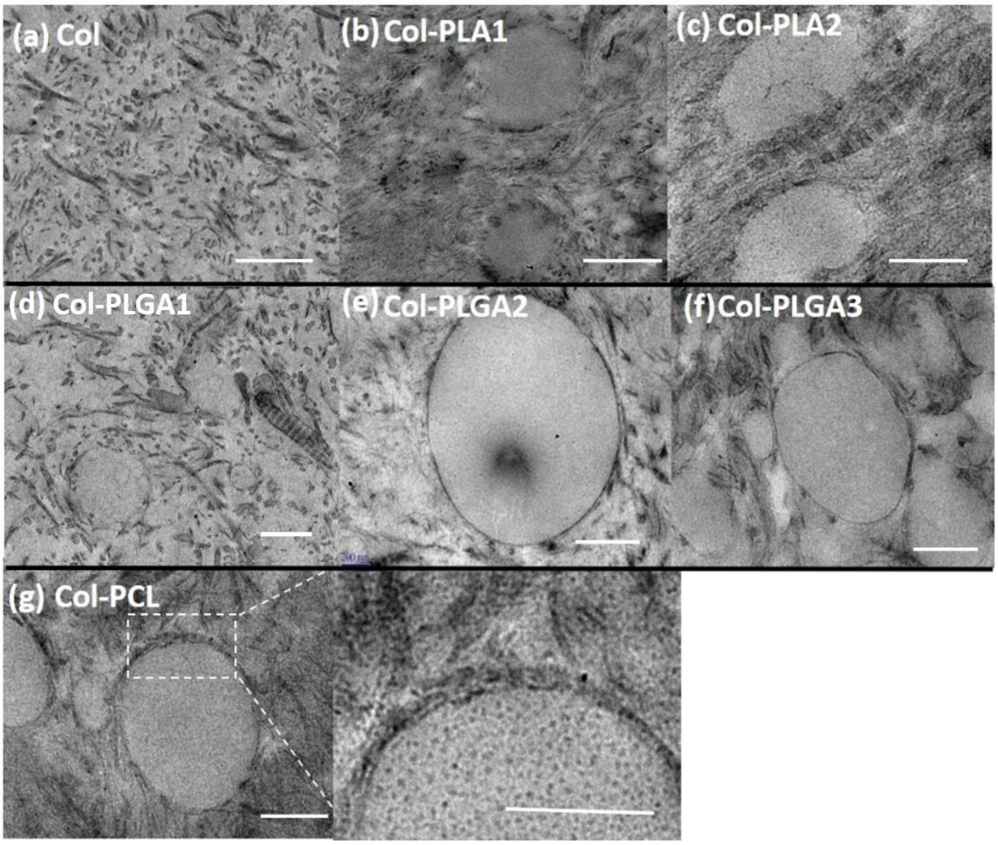

Figure S3. Structure of collagen composites observed by TEM images of pure collagen (a) and collagen composites(b-g) showing the interface of collagen fibril and hydrophobic polyesters, scale bar=0.5 µm.

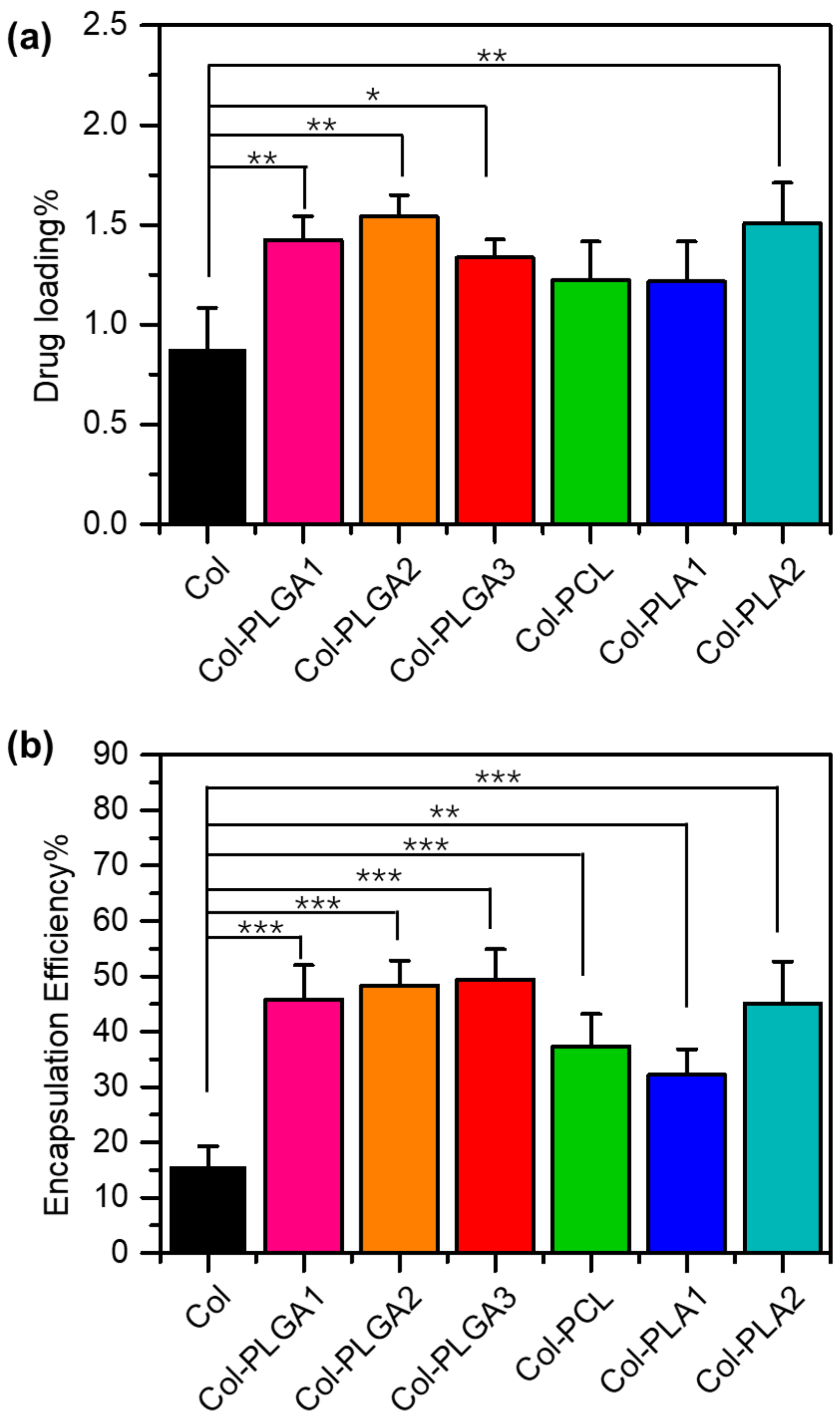

Figure S4. (a) Drug loading of spironolactone in pure collagen or the composites (n = 3). (b) Encapsulation efficiency of spironolactone in pure collagen or the composites (n = 3). Variance among all the groups was determined by one-way ANOVA with Dunnett's posthoc test (*P < 0.05, ** P < 0.01, *** P < 0.001).

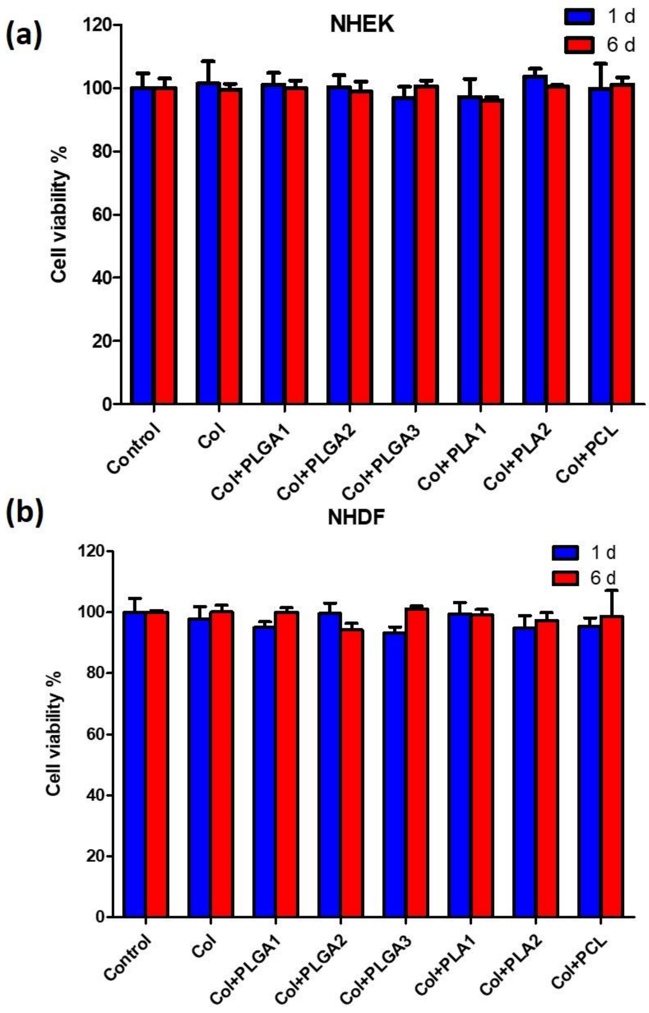

Figure S5. Cell viability of normal human epidermal keratinocytes (NHEK, a) and normal human dermal fibroblasts(NHDF, b) after 1 or 6 days in co-culture with non-drug loaded pure collagen or collagen composite hygrogels measured by Alamar blue assay (n=3). All groups exhibited cell viability around 100%, indicating excellent cytocompatibility of the collagen-polyester composites hydrogels prepared from in-situ nanoprecipitation method.

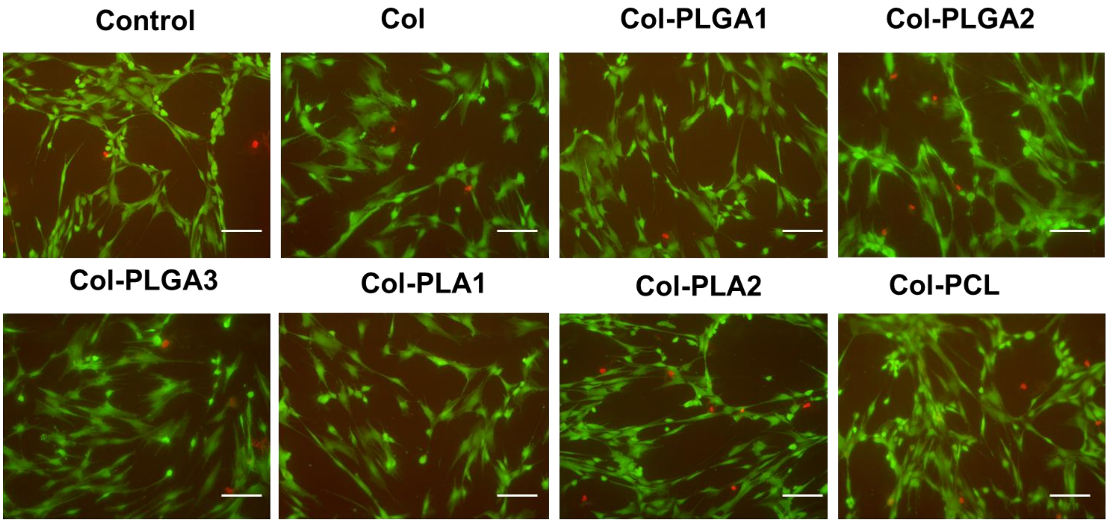

Viability of fibroblasts

Group	Control	Col	Col-PLGA1	Col-PLGA2	Col-PLGA3	Col-PLA1	Col-PLA2	Col-PCL
Percentage of living cells (%)	94.2±3.0	97.6±1.3	96.3±1.3	95.9±1.3	95.8±2.1	100±0.0	96.2±0.6	94.2±0.3

Figure S6. Viability of normal human dermal fibroblasts cultured with composite hydrogels for 24 hours measured by Live/Dead assay (Scale bar=50 μm).

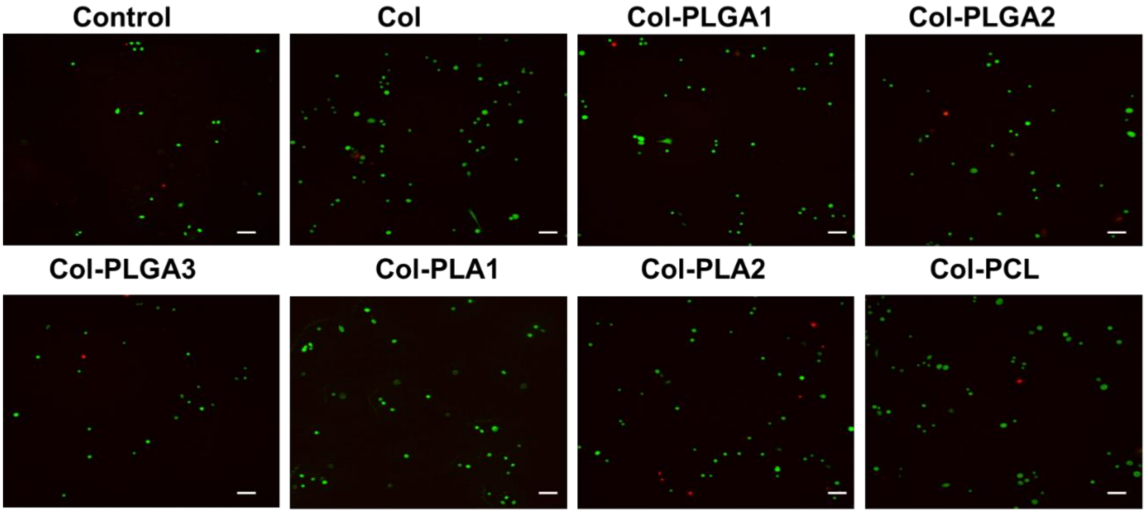

Viability of Keratinocytes

Group	Control	Col	Col-PLGA1	Col-PLGA2	Col-PLGA3	Col-PLA1	Col-PLA2	Col-PCL
Percentage of living cells (%)	98.2±1.7	98.4±0.6	99.8±0.3	99.6±0.6	99.2±0.5	97.1±3.0	98.9±1.0	99.4±0.7

Figure S7. Viability of normal human epidermal keratinocytes cultured with composite hydrogels for 24 hours measured by Live/Dead assay (Scale bar=50 μm).

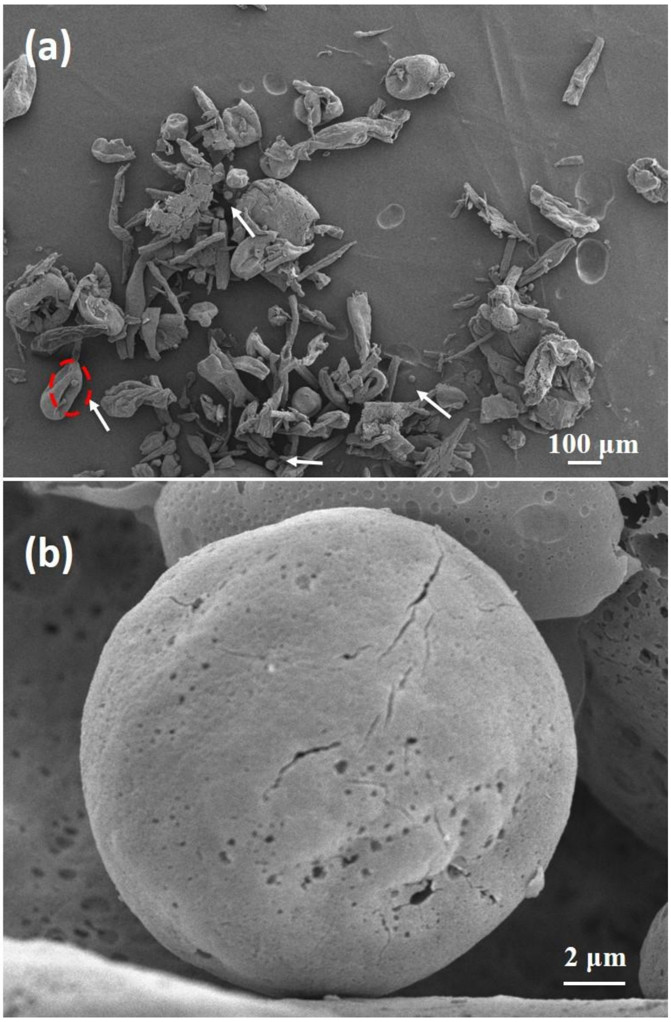

Figure S8. SEM images of precipites formed by pouring PLGA1 solution (160 mg.mL^{-1} in THF) fast into PBS (pH=7.4) at a volume ratio of 1:10, most of which were found in irrgular shape (a) and few were particles at size around 10 µm indicated by white arrows. (b) Enlargement of red-circle zone in (a) exhibited the morphology of microparticles formed in the precipitation process.

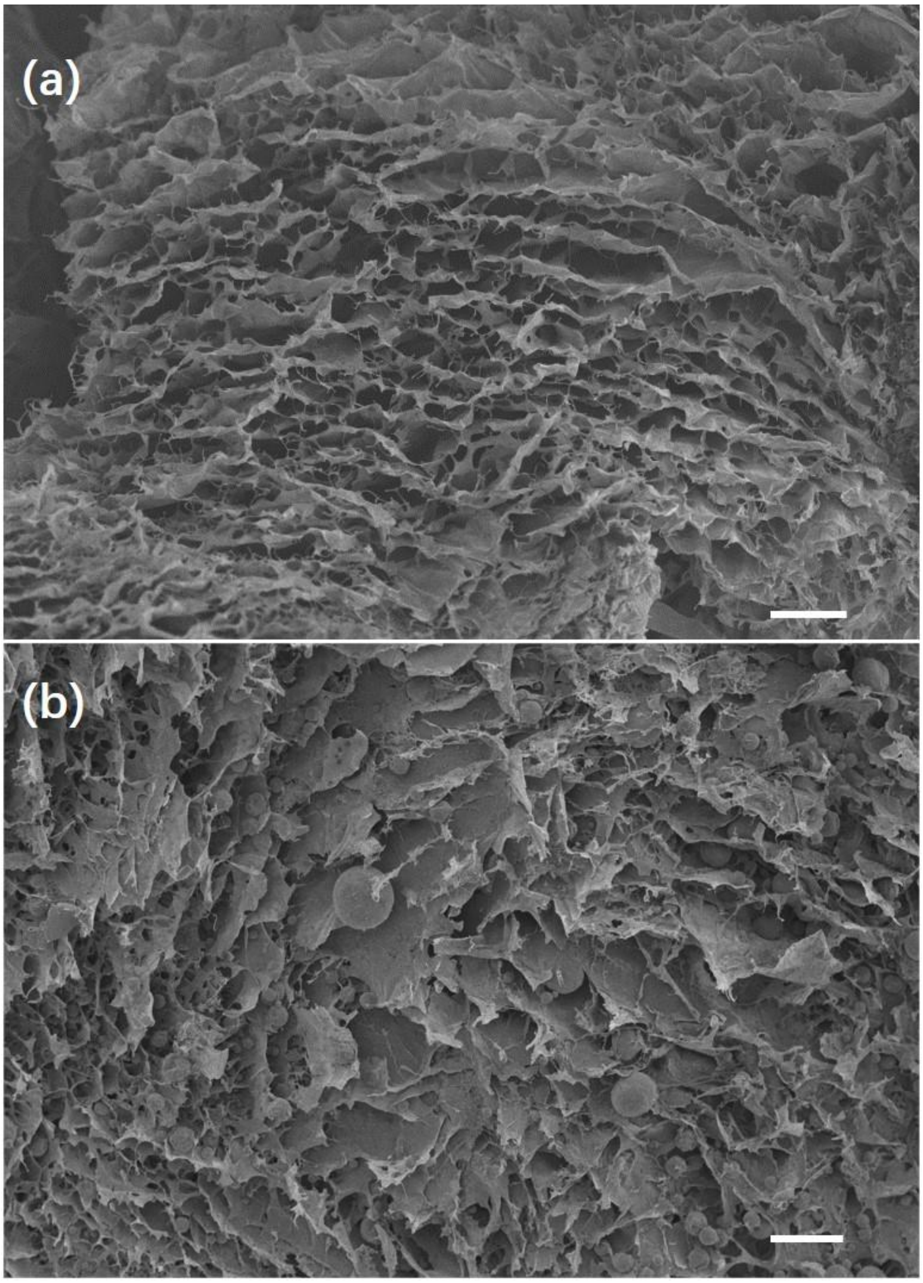

Figure S9. SEM image of (a)Alginate hydrogel prepared from alginate solution (40 mg.mL^{-1}) crosslinked by Ca^{2+} and (b)Alginate-PLGA1 compostie hydrogel obtained by the in-situ nanoprecipitaion of PLGA1 in the 3D alginate network. As alginate hydrogel was much more porous than collagen counterpart, larger particles with a averagely size around 5 µm were formed, i.e around 5-10 fold that of formed particles in collagen hydrogels, indicating the nature of hyrogel matrix plays a potent role in particle size controlling (Scale bar 50 µm).

References

[1] S. Farah, D. G. Anderson, R. Langer, *Adv. Drug Deliv.Rev.* **2016**, 107, 367.
